# A Sandwich HIV p24 Amperometric Immunosensor Based on a Direct Gold Electroplating-Modified Electrode 

**DOI:** 10.3390/molecules17055988

**Published:** 2012-05-18

**Authors:** Lei Zheng, Liyong Jia, Bo Li, Bo Situ, Qinlan Liu, Qian Wang, Ning Gan

**Affiliations:** 1 Clinical Laboratory Center, Nanfang Hospital, Southern Medical University, Guangzhou 510515, China; Email:nfyyzl@163.com (L.Z.); jialiyong@126.com (L.J.); boli198777@163.com (B.L.); drstb@126.com (B.S.); liuqinlan1206@163.com (Q.L.); 2 The State Key Laboratory Base of Novel Functional Materials and Preparation Science, Faculty of Material Science and Chemical Engineering of Ningbo University, Ningbo 315211, China

**Keywords:** HIV, p24, sandwich amperometric immunosensor, direct electroplating

## Abstract

Acquired immune deficiency syndrome (AIDS) is a severe communicable immune deficiency disease caused by the human immune deficiency virus (HIV). The analysis laboratory diagnosis of HIV infection is a crucial aspect of controlling AIDS. The p24 antigen, the HIV-1 capsid protein, is of considerable diagnostic interest because it is detectable several days earlier than host-generated HIV antibodies following HIV exposure. We present herein a new sandwich HIV p24 immunosensor based on directly electroplating an electrode surface with gold nanoparticles using chronoamperometry, which greatly increased the conductivity and reversibility of the electrode. Under optimum conditions, the electrochemical signal showed a linear relationship with the concentration of p24, ranging from 0.01 ng/mL to 100 ng/mL (R > 0.99), and the detection limit was 0.008 ng/mL. Compared with ELISA, this method increased the sensitivity by more than two orders of magnitude (the sensitivity of ELISA for p24 is about 1 ng/mL). This immunosensor may be broadly applied to clinical samples, being distinguished by its ease of use, mild reaction conditions, guaranteed reproducibility, and good anti-interference ability.

## 1. Introduction

Acquired immune deficiency syndrome (AIDS) is a severe communicable immune deficiency disease caused by the human immune deficiency virus (HIV). According to serological reactions, HIV is categorized into HIV-1 and HIV-2, which differ largely in their nucleotide sequences. However, HIV-2 is largely restricted to some regions of Western Africa and HIV-1 is found in most HIV strains worldwide, including China. There are still no efficacious therapeutic measures. Prevention of infection is the primary strategy to control HIV. The analysis laboratory diagnosis of HIV infection is a crucial aspect of controlling AIDS. Therefore, a sensitive and practical detection method to monitor, diagnose, and screen HIV infection is especially important for controlling AIDS. 

Currently, common methods for screening HIV infection in clinical practice include enzyme-linked immune sorbent assay (ELISA) based on a color reaction and quantitative fluorescence polymerase chain reaction (PCR) based on nucleotide amplification. ELISA is an accurate and high-performance method, but has some disadvantages such as tedious procedures, high-volume sample consumption, a long time requirement, low sensitivity, and a long detection “window phase” (see next paragraph). Therefore, it cannot be conducted within a short time in units with a rapid personnel flow (blood stations and clearance ports). Real-time PCR is an ideal quantitative method for diagnosing HIV, but high cost and complexity limit its use to primary medical institutions, especially in distal regions. Furthermore, in special situations, especially before emergency surgery, patients should be screened rapidly, but conventional methods are not capable of meeting the requirements due to their low sensitivity and specificity, time consumption, and complicated operations. For reasons of privacy and the fear of discrimination, high-risk populations are often willing to test themselves but not to go to hospital or centers for disease prevention. Consequently, a simple, rapid, sensitive, specific, and inexpensive HIV screening technique and corresponding portable instruments are required, and would be of great significance in preventing and controlling AIDS propagation. 

HIV p24 antigens appear at an earlier stage of HIV infection than antibodies, which is due to an explosive replication of the virus following acute infection and is correlated with highly infectious viraemia. Early detection of HIV p24 would be of great value in the early detection of HIV infection, blood screening, neonatal HIV infection, and the surveillance of therapeutic efficacy and disease progression. However, after acute HIV infection, specific antibodies occurring in the body combine with p24 antigens to form immune complexes, causing the free antigen concentration to become too low to be detectable, this period is called “window phase”. Although it cannot be detected during this period, there is already HIV existing in the body and can be passed on to others. Therefore, there is a need to develop a novel highly sensitive method to directly identify p24 antigens at window phase. 

The detection of p24 proteins is mostly conducted by ELISA. The fourth-generation HIV antibody ELISA detection kits that are currently in widespread use can also detect p24 protein. We have conducted several pilot studies on electrochemical immunoassay of p24 proteins [1]. However, most of these methods have been based on a “one-step” protocol, based on the nonconductive property of proteins. When antigens are captured by antibodies on the surface of electrodes, they will form an immune complex, which can block electron transfer on the electrode surface, so the current will decay, and the more immune complex formed, the larger the current decay. Detecting the current decay can provide a quantitative analysis the target proteins. In such an approach, detection errors arising from protein loss or inactivation as well as non-specific adsorption are inevitable, and the linear range is narrow. In recent years, sandwich amperometric immunosensors based on the ELISA principle have been developed [2,3,4,5,6]. In thid detection scheme, the primary antibody and enzyme (such as horseradish peroxidase [HRP])-labeled signal antibody recognize the antigen together to produce a “sandwich”-type immune complex. However, the signal generated from the enzyme-labeled antibody can catalyze a specific substrate, whose signal increases with an increase in the antigen concentration. Thus, the concentration of antigen can be determined by this method. Such sensors show significantly higher sensitivity and specificity than those based on the principle of “one-step immunoassay” and are a current hot topic in the field of electrochemical immunoassay [6,7,8,9,10,11,12]. On the basis of our pilot studies, an electrode surface was directly electroplated with gold nanoparticles using chronoamperometry, which greatly increased its conductivity and reversibility. Such gold nanoparticles not only provide a large surface area for immobilisation of a substantial quantity of antibodies, but they are also known to maintain the biological activity of antibodies [11,13,14,15]. In this way, capture anti-p24 monoclonal antibodies were adsorbed on an electrode surface and the remaining unconjugated active sites were blocked with bovine serum albumin (BSA). The capture antibody on the sensor was then allowed to interact with p24 antigen, and then the sensor was washed to remove the unreacted and/or nonspecifically adsorbed antigens after incubation. The electrode was then reacted with HRP-labeled signal antibodies (HRP-anti-p24) to form a sandwich immune complex (anti-p24/p24/HRP-anti-p24) on its surface. Thereafter, the electrode was placed in a solution containing hydrogen peroxide and hydroquinone. The HRP in HRP-anti-p24 catalyzed the reaction of hydrogen peroxide and hydroquinone. This was the basis of our quantitative analysis. Direct electroplating with gold nanoparticles using chronoamperometry is a simple, convenient method. By maintaining a constant electroplating time, we were able to obtain a relatively constant background current among different electrodes, give rise to acceptable reproducibility for mass production of this kind of sensors, all of which could make clinical application of this sensor come true.

## 2. Results and Discussion

### 2.1. Electrochemical Behavior of the Immunosensor

**Figure 1:**
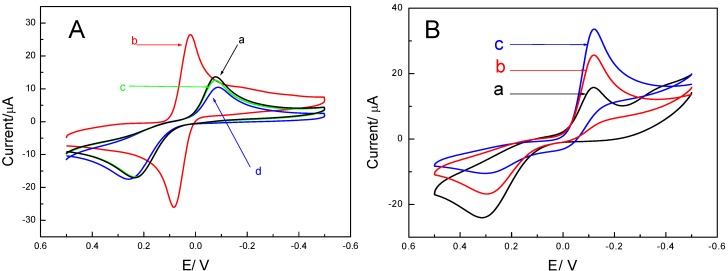
(**A**) Cyclic voltammograms of (a) bareGCE, (b) Au/GCE, (c) Ab1/Au/GCE, (d) BSA/Ab1/Au/GCEin pH 7.0 0.1 M PBS containing 1 mM hydroquinone. (**B**) Cyclic voltammograms of electrochemical immunosensor incubated with different concentration of antigen, (a) 0 ng/mL, (b) 20 ng/mL, (c) 50 ng/mL, and then incubated with signal antibody, in pH 7.0 0.1 M PBS containing 1 mM H_2_O_2_ and 1 mM hydroquinone.

Cyclic voltammetry is a simple and easy method to study the characteristics of the p24 antigen biosensor at its different preparation phases. Trace a in Figure 1A shows the cyclic voltammogram obtained at a bare electrode in 0.1 mol/L phosphate buffered saline (PBS) containing 1 mM hydroquinone at pH 7.0. When the potential was scanned from −0.5 V to 0.5 V, hydroquinone was oxidized to benzoquinone and an oxidation peak was observed at approximately 0.2 V. Similarly, when the potential was scanned from 0.5 V to −0.5 V, benzoquinone was reduced back to hydroquinone and a reduction peak was observed at approximately 0 V. When the same redox reaction was carried out at the Au/GCE, the cyclic voltammogram obtained (trace b) shows a 110% increase in peak current compared to that in trace a. This is attributable to the increase in surface area by the gold nanoparticles on the GCE. Similarly, a peak separation of 0.08 mV was observed in trace b compared to 0.3 mV in trace a, indicating an increased electrochemical reversibility of the hydroquinone/ benzoquinone reaction at the Au/GCE. When non-conducting anti-p24 antibodies were attached to the electrode surface, oxidation of hydroquinone and reduction of benzoquinone were hindered. As shown in Figure 1A-c, compared with the Au/GCE, the oxidation and reduction peaks both decreased by 55%, and the potential peak separation increased from 0.08 mV to 0.3 mV (see Figure 1), suggesting reduced electrode conductivity and reversibility. This also indirectly indicated successful deposition of anti-p24 antibodies on the electrode surface. The conductivity and reversibility were further diminished by 8% after blocking of the remaining binding sites with BSA (Figure 1A-d).

Figure 1B displays the changes in oxidation and reduction currents associated with the enzyme-catalyzed reaction before and after the formation of immune complexes on the electrode surface. Figure 1B-a shows the cyclic voltammograms of modified electrode (incubated with 0 ng/mL p24 antigen and then incubated with signal antibody, for the method see Section 3.2) in pH 7.0 PBS containing 1 mM hydroquinone and 1 mM hydrogen peroxide. Figure 1B-b shows the cyclic voltammogram of an electrode incubated with 20 ng/mL p24 antigen and then incubated with signal antibody under the same conditions. It can be seen that the reduction current was higher by 31% and the oxidation current was significantly lowered by 39% in Figure 1B-b than in Figure 1B-a, which was due to HRP on the electrode surface being able to catalyze the reaction of hydrogen peroxide and hydroquinone. The enzyme accelerated the catalytic reaction, which was manifest in an elevation of the reduction peak. Figure 1B-c shows the cyclic voltammogram of an electrode incubated with 50 ng/mL of p24 standard antigen. The reduction peak was further increased, while the oxidation peak was further decreased, showing the “up-shifting” trend. The increase in the reduction peak stemmed from HRP catalyzing the reaction of hydroquinone and peroxide. The magnitude of this increase was correlated with the quantity of enzyme fixed on the electrode, which was in turn correlated with the quantity of signal antibodies on the electrode surface (the quantity of immune complexes) and directly with the p24 concentration. Therefore, p24 could be quantified through the reduction currents change (ΔI) between the initial electrode (I_0_) and the electrode with the sandwich immune complex (I): ΔI = I − I_0_, using the ΔI to quantify the p24 can reduce the influence of background current.

**Figure 2:**
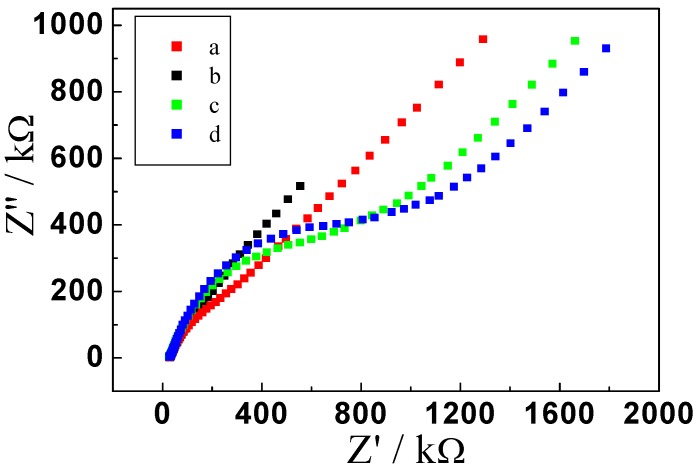
EIS of (a) bare GCE, (b) Au/GCE, (c) Ab1/Au/GCE, (d) BSA/Ab1/Au/GCE in 10 mmol/L Fe(CN)_6_^3−/4−^ containing 0.1 mol/L KCl, and the frequency range used was 0.1 to 1 × 10^5^ Hz.

Electrochemical impedance spectroscopy (EIS) is an effective method of representing the characteristics of modified electrode surfaces. The spectrum of alternating current (AC) consists of high- and low-frequency regions. The semicircle of the high-frequency region directly expresses the electron-transport impedance, the longer the diameter, the greater the impedance. We experimentally determined the EIS of the modified electrodes after the different steps. Figure 2 shows the Nyquist plots of EIS of the bare and modified electrodes. The curve (a) shows EIS of the bare GCE. There is a small semicircle at high frequencies and a linear part at low frequencies. When electroplate gold on the GCE surface, a lower resistance was obtained (b), implying that the AuNPs accelerated the electron transfer between the redox probe and the electrode. The resistance significantly increased after incubation with anti-p24 (c), suggesting that anti-p24 were successfully immobilized on the surface and blocked the electron exchange between the redox probe and the electrode. The electrical resistance of the sensor increased further when the remaining active sites on working electrode were blocked with 3%BSA (d). So, the EIS results were in accordance with those of cyclic voltammetry. 

**Figure 3:**
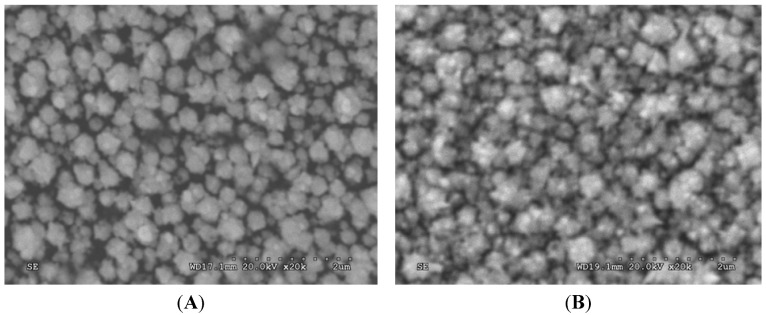
Scanning electron microscope images of (a) Au/GCE and (B) Ab1/Au/GCE.

In order to further confirm the assembly of nano-Au and anti-p24 on the electrode, scanning electron micrographs (SEMs) of Au/GCE and anti-p24/Au/GCE was recorded. As shown in Figure 3A, we can see that gold nanoparticles were tiny granules with approximate diameters of 300 nm, with some stabs and a dense array on the surface of the electrode. After the capture antibody was modified on gold nanoparticles, the SEM image of anti-p24/Au/GCE (Figure 3B) clearly shows the presence of numerous bright particles, indicating that antibody was successfully modified on the Au/GCE.

### 2.2. Optimization of Immunoassay Conditions

#### 2.2.1. Effect of Substrate Concentration on Immunoassay

**Figure 4:**
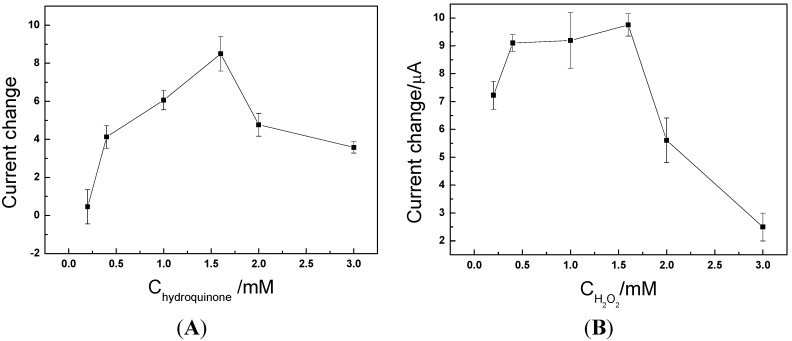
(**A**) Effect of hydroquinone concentration. (**B**) Effect of H_2_O_2_ concentration.

The effect of substrate concentration on the reduction peak current was investigated. The plot in Figure 4 shows that 1.5 mM hydroquinone and 1.5 mM hydrogen peroxide were the optimal reaction concentrations. We speculate that, as we use ΔI = I − I_0_ as the quantitative index, before the enzyme is saturated (C_hydroquinone_ and C_H2O2_ < 1.5 mM), the response rate increases with the concentration of substrate. Both I_0_ and I increased. Moreover, I increased at a faster rate than I_0_ so ΔI increased; when the enzyme was saturated, the response rate (I) remained unchanged and no longer increased, while I_0_ still increased so ΔI decreased.

#### 2.2.2. Effect of Buffer pH on the Sensor Operation

**Figure 5:**
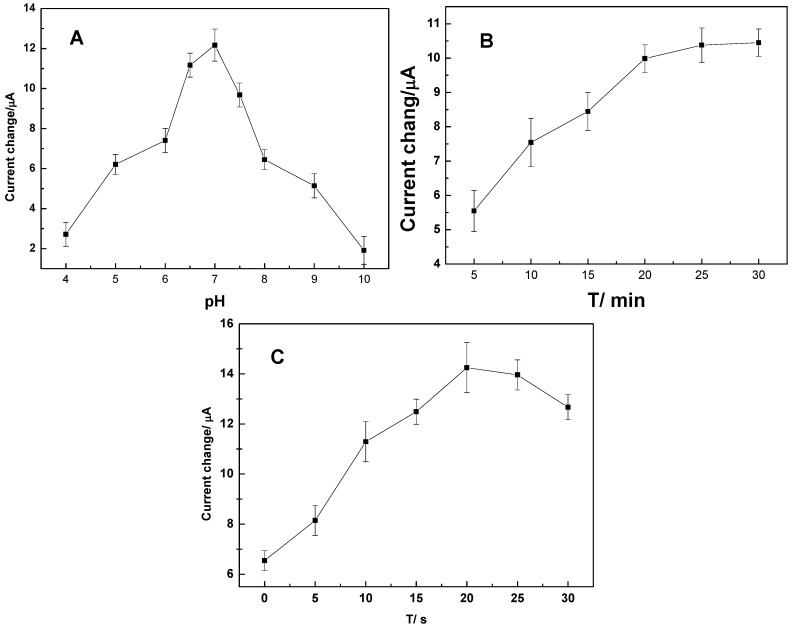
Effects of pH (**A**), incubation time (**B**), and electroplating time (**C**) on the response of the immunosensor.

The chemical reactions on electrode are: (1) immune response between p24 and anti-p24; (2) The HRP in HRP-anti-p24 catalyzed the reaction of hydrogen peroxide and hydroquinone. So the influence of pH on immunosensor operation was manifest in the direct effect both on immune response and HRP activity. Figure 5(A) shows changes in the peak current of the electrode before and after incubation with the antigen at different pH values. From Figure 5(A), it is evident that the peak current showed the greatest changes at pH 7.0, because that, as we speculated: (1) the isoelectric point of p24 is pH 6.7, which is very close to 7.0, when pH is 7.0, the immune responserate is highest; (2) the HRP have the best activity when the pH is 7.0.

#### 2.2.3. Effects of Incubation Time and Temperature on Sensor Operation

Experiments showed that the immunosensor displayed an excellent current response signal at room temperature. Therefore, analyses could be simply performed at room temperature. As shown in Figure 5(B), the catalytic current first increased with time and then became stable after 20 min, suggesting that immunological reactions on the electrode surface had been accomplished. Therefore, 20 min was chosen as the optimal time.

#### 2.2.4. Effect of Electroplating Time on Sensor Operation

As shown in Figure 5(C), the change in catalytic current reached its maximum at an electroplating time of 20 s and gradually dropped when electroplating was continued for a further 20 s. This may duo to that, the Au/GCE surface area increase within the first 20 s of electroplating, but then the nanoparticles connected to each other during the next 20 s to reduce the electrode surface area. We alsofound that, we can get a very closed background current (coefficient of variation <1%) even by using different electrodes, at different time. This inspired us to believe that this kind of sensor can be mass produced and easily applied in the clinic.

### 2.3. Detection of p24 by the Amperometric Immunosensor

**Figure 6:**
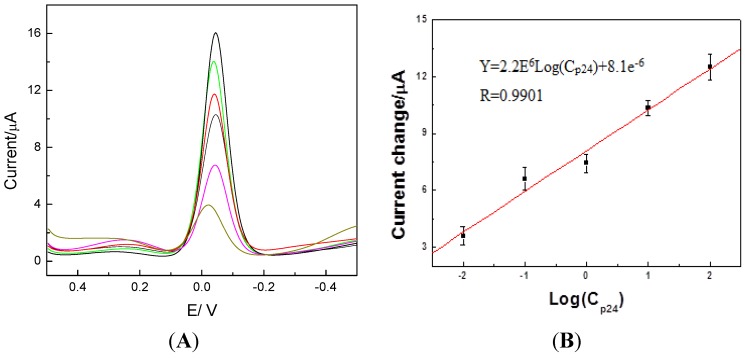
(**A**) Differential pulse voltammetric (DPV) curve. (**B**) Calibration plot of the immunosensor towards different concentrations of p24.

According to the optimization experiments, the detection conditions were as follows: buffer solution at pH 7.0, incubation time: 20 min at room temperature, direct gold electroplating time: 20 s, and concentrations of hydroquinone and peroxide: 1.5 mM. Differential pulse voltammetric (DPV) is more sensitive than cyclic voltammetry [3,15,16,17,18]. Therefore, the DPV method was applied in this study to detect p24 antigen in samples. In Figure 6, it can be seen that the current change showed a linear relationship with the p24 concentration in the range 0.01-100 ng/mL, with a linear relationship coefficient of 0.9901. We detected blank solution 10 times and get the lower detection limit was 0.008 ng/mL (3 times the standard deviation). Compared with 0.32 μg·L^−1^ in our previous study [19]. This sensor has a lower detection limit.

### 2.4. Reproducibility and Anti-Interference Ability and Stability of the Immunosensor

Serum from health donors obtained from Nanfang Hospital were added to 10 ng/mL and 50 ng/mL HIV antigen and then were each assayed three times with different batches of immunosensors prepared at different times. The interclass relative standard deviations were 2.5% and 3%, respectively, displaying excellent precision. The main interfering species in the serum were substances with electric activity such as ions, dopamine, L-cysteine, and uric acid. Our studies indicated that the above substances had no effect on detection (<5%), even at 10-100 fold higher concentrations in the serum, illustrating robust anti-interference ability. The proposed sensor can be stored in pH 6.5 PBS (4 °C) for 45 days, and its signal did not have obvious changes (<7%) which showed the sensor to have good storage stability. Electrode can be refreshed to use, the measured results of the70 ng/mL and 15 ng/mL p24 sample were, respectively, 67.69 and 15.7 ng/mL and RSD (n = 3) was, respectively, 3.3% and 3.2%.

### 2.5. Detection of HIV in Samples with the Immunosensor

HIV content in human serum was assayed by the developed method. The serum sample was diluted 1-20 times with 0.1 mol/L pH 7.0 PBS without other treatments. HIV signals detected in three samples were consistent with the results by ELISA (*p *> 0.05). The recovery of a standard addition was 95-110%.

**Table 1:** Results of p24 determinations in human serum.

Sample	HIV concentration (ng/mL)				
	Immunosensor	ELISA	Addition	Result	Recovery rate % ^a^
1	5.3	5.2	5.0	10.2	98
2	9.8	10.3	9.0	18.5	95.6
3	20.5	21.3	20.0	41.7	106

^a^ Recovery = 100% × (measurement of addition − measurement of sample)/addition.

## 3. Experimental

### 3.1. Instruments and Reagents

Hydroquinone (HQ) and H_2_O_2_ were purchased from Shanghai Crystal Pure Reagent Co., Ltd. (Shanghai, China). HIV monoclonal antibody solution (Ab_1_, Clone number:39/5.4A) and HIV ELISA kits were obtained from Abcam (Cambridge, MA, USA), which contain p24 stand protein and HRP-anti-p24; BSA was purchased from Sigma-Aldrich (St. Louis, MO, USA), 0.1M PBS as buffer solution and blank solution was prepared in our lab, add HCl or NaOH in to obtain different pH PBS. All other reagents were of analytical grade. Doubly-distilled water was used for all experiments.

Cyclic voltammetric measurements were performed on a CHI 660d electrochemistry workstation (Shanghai CH Instruments, Shanghai, China) with a three-electrode system composed of a platinum wire auxiliary electrode, a saturated calomel reference electrode (SCE), and a 2 mm bare or modified glassy carbon electrode (GCE; purchased from Shanghai CH Instruments) as a working electrode. Scanning electron microscope images were obtained by S-3400 (Hitachi, Tokyo, Japan).

### 3.2. Methods

#### 3.2.1. Preparation of a Sandwich HIV p24 Amperometric Immunosensor Based on Gold Nanoparticle-Modified Electrode

**Scheme 1:**
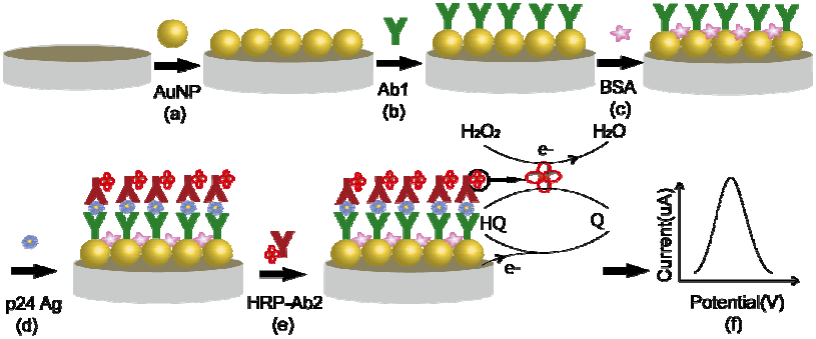
Preparation procedures of the immunosensor and detection principle.

Scheme 1 shows the procedures involved, which are outlined in the following steps: (a) Direct gold electroplating on the surface of a glassy carbon electrode (GCE). After polishing, the electrode was immersed in a solution, containing 1 mg/mL of tetrachlorauric(III) acid and 1 mM sulfuric acid. Direct gold electroplating on the surface of the GCE was conducted using chronaoamperometry by applying a reduction voltage at −0.2 V. The electrode was washed with ultrapure water to remove loosely bound gold particles and allowed to dry in air. A gold nanoparticle-modified electrode (Au/GCE) was thereby obtained. (b) Immobilisation of anti-p24 monoclonal antibodies (capture antibodies): Anti-p24 monoclonal antibodies were immobilized on the Au/GCE by direct adsorption. Anti-p24 monoclonal antibodies (10 μL, 10 μg/mL) were applied to the electrode by means of a volumetric micropipette and the system was incubated at 4 °C overnight. The electrode bearing the capture antibody is denoted as Ab1/Au/GCE. (c) Blocking with BSA: The electrode was carefully washed with phosphate buffered saline (PBS) to remove excess antibodies and then left to dry (30 min). It was subsequently incubated with 3%(v/v) BSA at 37 °C for 1 h, then thoroughly washed with PBS and dried, and finally stored in a refrigerator at 4 °C. 

#### 3.2.2. Scanning Electron Microscopy (SEM)

The morphology and particle size of gold nanoparticle on the modified electrode before and after capture antibody loading was examined with scanning electron microscopy S-3400 (Hitachi, Tokyo, Japan) in the State Key Laboratory Base of Novel Functional Materials and Preparation Science, Faculty of Material Science and Chemical Engineering of Ningbo University. The SEM was conducted at 10.0 kv.

#### 3.2.3. Detection Procedures of the Electrochemical Immunosensor

The preparation method of the p24 amperometric immunosensor in this study was similar to the double-antibody sandwich ELISA method. The principle of the electrode detection is shown in Scheme 1.

The procedures involved: (d) Incubation of the prepared electrodes with standard p24 antigens or serum (37 °C, 30 min). p24 antigens and anti-p24 antibodies on the surface of the electrodes reacted immunologically to form immune complexes. The electrode was then carefully washed with PBS. (e) The resulting electrode was immersed in a solution containing HRP-anti p24 antibodies at 37 °C for 30 min and then carefully washed with PBS. (f) The electrode was placed in a solution containing hydrogen peroxide and hydroquinone for 5 min. Differential pulse voltammetry (DPV, −0.5 V to 0.5 V, pulse width is 0.2 s, pulse amplitude is 0.05 V) was applied in the assay. The change in the reduction current of benzoquinone as a function of p24 antigen concentration was measured. 

## 4. Conclusions

Although electrochemical methods for the assay of p24 antigen have been reported previously, most of them are based on a “one-step” protocol [20,21,22], that is to say, a current-decay method, making them inevitably susceptible to false positive readings induced by protein inactivation. In this study, we have successfully constructed a sandwich HIV p24 amperometric immunosensor using a gold electroplating-modified electrode. Nano-gold was electroplated on the GCE surface using chronoamperometry in order to increase the conductivity and reversibility of the electrode. The nano-gold could also fix massive antibodies and maintain their biological activity. Reaction between the antigen and the secondary antibody formed a sandwich immune complex on the electrode surface. HRP in the signal antibodies catalyzed the reaction of hydroquinone and peroxide, which was manifested in a decrease in the oxidation current and an increase in the reduction current. This sensor displayed an excellent linear response to p24 within the concentration range 0.01-100 ng/mL. Compared to ELISA, this method increased the sensitivity by more than two orders of magnitude (the sensitivity of ELISA for p24 is about 1 ng). The method proved to be simple and reliable, with possible application in clinical HIV-positive samples. Compared with previous studies [1,19], our sensor has a lower detection limit. Direct electroplating with gold nanoparticles gold nanoparticles using chronoamperometry is a simple and convenient method. By maintaining a constant electroplating time, we were able to obtain a relatively constant background current among different electrodes, giving rise to an acceptable reproducibility for mass production of the sensor, all of which make clinical application of this sensor a potential fact.
